# No Correlation Between Chronic Cough and Radiographic Signs of Bronchial Narrowing in Dogs with Cardiomegaly and Left Atrial Dilation Secondary to Primary Mitral Valve Regurgitation

**DOI:** 10.3390/ani15172510

**Published:** 2025-08-26

**Authors:** Kira Y. van Opstal, Mark D. Kittleson, Erik Teske, Edoardo Auriemma, Henk van den Broek, Giliola Spattini, Federico R. Vilaplana Grosso, Viktor Szatmári

**Affiliations:** 1 Department of Clinical Sciences, Faculty of Veterinary Medicine, Utrecht University, 3584 CM Utrecht, The Netherlands; 2 Veterinary Information Network and School of Veterinary Medicine and Epidemiology, University of California, Davis, CA 95616, USA; 3 AniCura Istituto Veterinario Novara, 28060 Granozzo con Monticello, NO, Italy; 4 Clinica Veterinaria Castellarano, 42014 Castellarano, RE, Italy; 5 Department of Small Animal Clinical Sciences, College of Veterinary Medicine, University of Florida, Gainesville, FL 32611, USA

**Keywords:** bronchomalacia, bronchial collapse, bronchial compression, bronchitis, bronchoscopy, chronic mitral valve disease, fluoroscopy, furosemide, left mainstem bronchus, murmur, myxomatous mitral valve degeneration, pimobendan, torasemide, tracheal collapse, tracheobronchomalacia

## Abstract

Background: Some dogs with severe mitral valve regurgitation can have chronic cough, but its cause is unclear. Many of these dogs exhibit left principal (synonym mainstem) bronchial collapse or compression. Some clinicians believe that an enlarged heart, particularly the dilated left atrium, compresses the left principal bronchus, while others argue that a healthy bronchus cannot be compressed and that the cough is due to concurrent airway disease, such as bronchomalacia. In this study, we aimed to investigate the correlation between chronic cough and radiographic signs of bronchial narrowing in dogs with cardiomegaly secondary to primary mitral valve regurgitation. Methods: Four radiologists independently evaluated 51 sets of thoracic radiographs from client-owned dogs with cardiomegaly due to severe mitral valve regurgitation, including 26 sets of radiographs belonging to dogs with chronic (>8 weeks) cough and 25 without cough. The presence or absence of bronchial narrowing was assessed while the radiologists were blinded to patient characteristics and each other’s findings. Results: Interobserver variability was high, and the radiologists could not reliably predict which dogs had a chronic cough. Dogs with left atrial enlargement and airway narrowing sometimes coughed and sometimes did not. Likewise, some dogs with left atrial enlargement but no signs of airway compression or collapse coughed, and some did not. It was impossible to distinguish the groups based on these features. Conclusions: Bronchial compression by an enlarged heart and left atrium is an unlikely cause of chronic cough in dogs with severe primary mitral valve regurgitation.

## Simple Summary

Mitral valve regurgitation (MR) is the most common heart disease in dogs, particularly affecting middle-aged to elderly toy and small breeds. Severe MR leads to enlargement of the heart, especially the left atrium, which on radiographs often appears to compress the adjacent airways. Chronic cough, frequently due to chronic bronchitis and/or airway collapse, is also common in these dogs. The cause of coughing in dogs with a severely enlarged left atrium remains debated. Some experts attribute the cough to mechanical compression of the airways by the enlarged heart, while others believe that healthy airways cannot be compressed and that the cough is due to concurrent airway disease, with airway collapse secondary to mural weakness. Our study aimed to investigate the correlation between chronic cough, severe left atrial enlargement, and airway narrowing. Four independent veterinary radiology specialists evaluated 51 sets of radiographs for evidence of bronchial narrowing from coughing and non-coughing dogs, all with left atrial enlargement. None of the radiologists could reliably predict which dogs had a chronic cough, and agreement on bronchial narrowing was poor. Dogs with left atrial enlargement and radiographically evident airway narrowing sometimes coughed and sometimes did not. Similarly, some dogs with left atrial enlargement but no apparent airway narrowing coughed, and some did not. It was impossible to distinguish the groups based on these radiographic features. We conclude that bronchial compression by an enlarged heart is an unlikely cause of chronic cough in dogs with left atrial enlargement.

## 1. Introduction

Approximately 10% of dogs examined by primary care veterinarians have heart disease, with myxomatous mitral valve degeneration (MMVD) causing mitral regurgitation (MR) being by far the most common form [1,2,3,4,5]. This condition primarily affects middle-aged to elderly small and toy breed dogs. Severe MR leads to severe cardiomegaly from chronic left ventricular and left atrial (LA) volume overload. The disease typically has a long subclinical phase (stages B1 and B2) that lasts several years before some dogs develop clinical signs of left-sided congestive heart failure (stage C), like tachypnea and dyspnea due to pulmonary edema [1,2,3,4].

Coughing is also frequently reported in dogs with varying degrees of MR, but its relationship with MR, specifically the severely enlarged LA, is unclear since these dogs are prone to both MR due to MMVD and chronic respiratory conditions like tracheobronchomalacia and chronic idiopathic bronchitis [1,6,7,8,9,10,11,12,13,14,15,16,17]. A common radiographic finding in these dogs with severe LA enlargement and a cough is narrowing (due to collapse or compression) of the left principal (synonym mainstem) bronchus. Two main theories exist for how this relates to the cough in these dogs. The first is that these dogs have concurrent respiratory disease [2,12,13,17]. In this scenario, the cough arises from a primary airway disorder, such as bronchomalacia, and so the apparent left principal bronchial collapse. This is based on the theory that healthy bronchi cannot be compressed by severe LA enlargement. The second theory is that the severely enlarged LA physically compresses the left principal bronchus, causing cough even without preexisting respiratory disease [2,4,14,15].

This study examined whether radiographic signs of left principal bronchial narrowing correlate with chronic cough in dogs with LA enlargement. The goal was to determine which theory better explains the etiology of cough. The null hypothesis was that there would be no distinguishing radiographic features of principal or lobar bronchial narrowing between coughing and non-coughing dogs with severe cardiomegaly. A secondary objective was to establish the interobserver variability between the radiologists related to identifying radiographic abnormalities and predicting coughing.

## 2. Materials and Methods

### 2.1. Case Selection

This retrospective observational study included dogs with severe radiographic cardiomegaly, defined as a vertebral heart scale (VHS) ≥ 11.5, and severe radiographic left atrial enlargement, defined as vertebral left atrial size (VLAS) ≥ 2.5 [1,5,18,19]. Cases were identified from the digital database of the Companion Animal Teaching Hospital at Utrecht University, the Netherlands, with the diagnosis of MMVD from the past 15 years. The presence or absence of chronic cough (defined as a duration of ≥8 weeks [6,11,12,13,20]) was recorded from the medical history, as reported by the owners. No active selection took place to a balanced coughing versus non-coughing ratio. Only dogs whose owners explicitly reported coughing were classified as such. During the history taking, the cardiologists always asked about the presence of cough, which reduces the chance for underreporting. Radiographs showing any apparent lung or pleural space disease that could impact bronchial evaluation, as described in the attending radiologist’s report, were not included.

Eligible dogs had to have at least two digital radiographs in orthogonal projections (one lateral and one dorsoventral), both acquired and archived at the same institution. The case selection process, including the measurement of VHS and VLAS, was performed by the first author, a final-year veterinary student.

Multiple sets of radiographs from the same dog were permitted, provided there was a minimum interval of three months between serial radiographs. A written report for all radiographs was available, prepared by a European Board of Veterinary Specialisation (EBVS)-recognized specialist, certified diplomate by the European College of Veterinary Diagnostic Imaging (ECVDI; further called “radiologist”).

### 2.2. Assessment of Radiographs

Four independent EBVS-recognized specialists in veterinary diagnostic imaging (radiologists, designated R1–R4) evaluated all thoracic radiographs. All four participating EBVS-recognized and ECVDI-certified radiologists completed their training at Utrecht University. Two obtained their diplomate status in 2008, one in 2015, and one in 2024. They specifically assessed decreased bronchial luminal diameter due to collapse or compression. The two principal bronchi (right and left) and the seven lobar bronchi (i.e., the right cranial, right middle, right caudal, accessory, cranial segment of the left cranial, caudal segment of the left cranial, and left caudal) were scored as follows: non-compressed/collapsed (0), compressed or collapsed with <75% lumen reduction (1), compressed or collapsed with >75% lumen reduction (2), or not visible/unable to interpret (X). The radiologists were not specifically instructed how to assess the radiographs for bronchial narrowing (i.e., compression/collapse).

Because decreased bronchial lumen can result from either compression or collapse (as seen in bronchomalacia), the term compressed/collapsed is used throughout the text.

The presence of abnormal lung patterns (interstitial, bronchial, or alveolar) was scored as present (1) or absent (0). Both VHS and VLAS were measured for each case. Each radiologist was also asked to predict whether the radiographic changes could (1) or could not (0) cause a chronic cough. If a chronic cough was considered possible, the presumed cause was specified (collapsed/compressed bronchus, lung pathology, or both).

This assessment generated 16 variables: 2 continuous (VHS and VLAS) and 14 categorical (scores for the two principal and seven lobar bronchi, three types of abnormal lung patterns, the radiologists’ predictions regarding chronic cough, and the presumed cause of cough). Cases were included if at least one radiologist reported a VHS ≥ 11.5 and a VLAS ≥ 2.5 [5]. Cases were excluded if all four radiologists reported a VHS < 11.5 or a VLAS < 2.5.

Prior to review, all digital radiographs were anonymized: all identifying text (including the dog’s signalment and name, owner’s name, date of birth, and date of radiograph) was digitally removed. The radiograph sets were then randomly ordered and numbered consecutively. Only the first author retained the key linking case numbers to specific dogs. Radiologists were blinded to signalment, medical history, clinical findings, and results of other imaging modalities (e.g., echocardiography, fluoroscopy). They were also unaware of the identities of other participating radiologists and blinded to one another’s interpretations. The proportion of coughing dogs among the cases was not disclosed.

Intraobserver variability was assessed by including two duplicate radiograph sets—one from a coughing dog and one from a non-coughing dog—selected for the absence of distinguishing features (e.g., collar, pacemaker, microchip). These duplicates were randomly interspersed within the evaluation sequence.

### 2.3. Statistical Analysis

#### 2.3.1. Power Analysis

Before the study, a power analysis was performed using G*Power (v3.1.9.6; Universität Düsseldorf, Düsseldorf, Germany), targeting a power of 0.8–0.9. The calculation, based on the prevalence of coughing in dogs with MMVD without concurrent left-sided congestive heart failure (CHF), indicated that 43–56 cases were needed (α = 0.05, effect size = 0.43).

#### 2.3.2. Descriptive Statistics and Contingency Table Analyses

Descriptive statistics were first calculated. To assess the association between chronic cough and left principal bronchus narrowing, contingency tables were constructed for each radiologist individually and for all radiologists combined (i.e., parallel testing), using free online software (MedCalc^®^ Software Ltd. Diagnostic Test Evaluation Calculator v23.2.1, Ostend, Belgium). Four variables were included: cough, no cough, bronchial compression/collapse, and no bronchial compression/collapse. Severe bronchial compression/collapse (i.e., >75% lumen reduction) was first tested against the combination of no and mild to moderate (i.e., <75% lumen reduction) bronchial compression/collapse. True positives were defined as dogs with severe bronchial compression/collapse that were coughing; false negatives as dogs with severe bronchial compression/collapse that were not coughing; true negatives as dogs with no or mild to moderate bronchial compression/collapse that were not coughing; and false positives as dogs with no or mild to moderate bronchial compression/collapse that were coughing. A secondary analysis tested the absence of bronchial compression/collapse against the combination of all levels (i.e., mild, moderate, and severe) of bronchial compression/collapse.

#### 2.3.3. Regression Models

Univariate analyses were performed for each radiologist to explore correlations between radiographic variables and both reported cough and the radiologists’ predictions of cough. Fisher’s exact test was used for the 12 categorical variables (bronchial and lung pattern scores), and univariate logistic regression was applied to the two continuous variables (VHS and VLAS). For bronchial scores, mildly to moderately compressed (1) and severely compressed (2) were combined to create binary variables. Statistical significance was set at *p* < 0.05. To address multiple comparisons, a Bonferroni correction was performed where appropriate. Subsequently, a multivariable logistic regression model was constructed for each radiologist using backward regression on the binary variables, abnormal lung patterns, and VHS and VLAS values to identify variables (alone or in combination) associated with reported cough and the radiologists’ predictions. Statistical analyses were performed using online software (Rstudio^®^, Posit team, Integrated Development for R, 2024.12.0+467, PBC, Boston, MA, USA; URL: https://posit.co; accessed 10 March 2025).

#### 2.3.4. Reliability Assessment

Interobserver variability among the four radiologists was assessed using the kappa statistics for the 14 categorical variables. Kappa scores < 0.40 were considered poor agreement, 0.40–0.60 moderate agreement, 0.60–0.80 good agreement, and >0.80 excellent agreement [21]. Fleiss’ kappa was also calculated to assess multi-rater agreement. The intraclass correlation coefficient (ICC) was used for the two continuous variables to measure total variation among radiologists; ICC < 0.50 was considered poor reliability, 0.50–0.75 moderate reliability, 0.75–0.90 good reliability, and >0.90 excellent reliability [22].

## 3. Results

### 3.1. Study Sample

A total of 59 radiograph sets were initially selected for review. Of these, 27 sets were from dogs with chronic cough, while 32 sets were from dogs that were either not coughing or had a cough of less than eight weeks’ duration at the time the radiographs were taken. These 59 sets originated from 44 individual dogs. The study sample included a variety of breeds, with Cavalier King Charles Spaniel being the most common (n = 18), followed by Chihuahua (n = 6), mixed-breed dogs (n = 4), Pomeranian (n = 3), Dachshund (n = 2) and King Charles Spaniel (n = 2). One of each of the following breeds was represented: English Cocker Spaniel, Beagle, Markiesje, Lion Dog, Whippet, Jack Russell Terrier, Miniature Pinscher, Griffon Bruxellois, and Coton de Tuléar.

The median age of the dogs was 9 years and 11 months, with a range from 5 to 15.5 years. The median body weight was 9.1 kg, ranging from 2.2 to 24.4 kg. As determined by the first author during case selection, the median VHS was 12.3 (range 11.5 to 14.0), and the median VLAS was 2.9 (range 2.5 to 4.1).

All dogs were client-owned and had been referred to the cardiology service of the Companion Animal Teaching Hospital at Utrecht University for various reasons. In all cases, MR secondary to MMVD was considered the cause of LA enlargement and radiographic cardiomegaly. The diagnosis of MR due to MMVD was established either by echocardiography (n = 40, with 38 performed at Utrecht University by an EBVS-certified veterinary cardiologist and two performed elsewhere before referral) or, in a minority of cases (n = 4), based on signalment (i.e., middle-aged to old small breed dog) and murmur characteristics (i.e., systolic murmur with an intensity of at least three out of six with the point of maximal intensity at the level of the mitral valve). Dogs were either in stage B2 or in stage treated C.

Of the 59 dogs, 21 did not receive any medication, and the remaining dogs were treated with furosemide + benazepril + pimobendan (n = 2, one is coughing one is non-coughing), furosemide + pimobendan (n = 2, both coughing), furosemide + benazepril + pimobendan + dexamethasone (n = 1, coughing), furosemide + pimobendan + bromhexine (n = 1, coughing), furosemide (n = 2, both coughing), benazepril + spironolactone (n = 1, non-coughing), torasemide + pimobendan (n = 1, coughing), pimobendan + mexiletine + sotalol (n = 1, non-coughing), benazepril + furosemide (n = 2, one is coughing, one is non-coughing), benazepril + digoxin (n = 1, coughing), dexamethasone injection within a week of examination (n = 1, coughing), antitussive syrup (n = 1, coughing), and pimobendan only (n = 5). An additional 17 radiographs were from dogs that were participating in the EPIC study [23], a placebo-controlled, double-blinded study; therefore, it is unknown whether these dogs were treated with pimobendan or placebo when the radiographs were made. One of them (coughing) received furosemide in addition to the test drug.

### 3.2. Case Exclusions

Of the 61 radiograph sets sent to the radiologists (59 unique sets from 44 dogs and 2 duplicate sets for intraobserver analysis), 2 sets were excluded because all four radiologists determined that the VLAS was less than 2.5. An additional six sets were excluded due to clinical criteria: five sets belonged to dogs with a cough duration of less than eight weeks, and one set belonged to a dog for which the duration of cough was not clearly documented.

After these exclusions, 51 unique radiograph sets remained for the final analysis. Among these, 26 sets were classified as non-coughing and 25 as chronically coughing. Nine dogs contributed more than one radiograph set, reflecting serial imaging over time. Of these, five dogs consistently remained in the non-coughing category, two remained in the chronic coughing category, and three transitioned from the non-coughing to the chronic coughing group between imaging sessions. Four dogs contributed two sets of radiographs each (two Cavalier King Charles Spaniels, one Markiesje, and one Lion Dog), while five dogs (all Cavalier King Charles Spaniels) contributed three sets each. In total, the final analysis included radiographs from 37 individual dogs. The breed distribution remained similar, with Cavalier King Charles Spaniel (n = 15) still being the most represented breed.

### 3.3. Image Review

Of the 61 sets of radiographs reviewed, 9 were provided as high-quality JPEG files only, while the remaining 52 sets were available in both JPEG and DICOM formats. For 30 of the sets, additional projections beyond the standard left lateral and dorsoventral views were available. Of these 30 sets, 20 sets contained one additional (right) lateral projection, and for 4 sets, an additional ventrodorsal projection was available. Six sets contained 4 images (an additional image in both directions). All radiograph interpretations were performed in December 2024.

All four participating radiologists estimated the presence or absence of bronchial compression/collapse based on subjective assessment as luminal narrowing of the specific bronchus. One radiologist added that a comparison with other bronchi was performed as well, while another radiologist added that a comparison with the contralateral bronchus was performed where this was appropriate.

### 3.4. Sensitivity and Specificity of Bronchial Compression/Collapse as a Predictor of Cough

The sensitivity and specificity of bronchial compression/collapse as predictors of cough were calculated using contingency tables, as described in Section 2. Sensitivity was defined as the ability of radiographic bronchial compression/collapse to correctly identify dogs with cough, while specificity reflected the ability to correctly identify dogs without cough in the absence of these radiographic changes. The radiologists’ findings (Table 1) formed the basis for these calculations.

Notably, there was disagreement among the radiologists regarding the presence of left principal bronchus collapse, particularly with Radiologist 4 differing from the other three. Therefore, combined results were calculated both for all four radiologists and separately for Radiologists 1–3, excluding Radiologist 4’s assessments, to account for this interobserver variation.

For the left principal bronchus, sensitivity and specificity varied significantly among radiologists (Table 2). Radiologists 1–3 demonstrated low sensitivity (range: 20–35%) and moderate specificity (range: 55–65%) in identifying bronchial compression/collapse as a predictor of cough. In contrast, Radiologist 4 showed 0% sensitivity (failing to identify any coughing dogs with severe compression/collapse) but 100% specificity (correctly excluding all non-coughing dogs without severe changes, but only because this person did not think any left principal bronchus was severely compressed/collapsed).

When combining mild to moderate and severe bronchial compression/collapse into a single category (versus no compression/collapse), sensitivity improved across all radiologists (range: 45–70%), though specificity decreased by 15–25 percentage points compared to analyses isolating severe cases (Table 2).

Using combined data from all four radiologists, severe left principal bronchial compression/collapse exhibited a sensitivity of 0.60 (60% of coughing dogs correctly identified) and a specificity of 0.58 (58% of non-coughing dogs correctly excluded). Exclusion of Radiologist 4’s outlier results improved specificity to 0.65 while maintaining sensitivity at 0.60, suggesting greater diagnostic consistency among the remaining three observers (Table 2).

Radiologists 1, 2 and 3 were able to interpret the left principal bronchus for compression/collapse in all cases, and Radiologist 4 reported one case where this bronchus was non-interpretable.

For the left caudal lobar bronchus, sensitivity for detecting cough based on severe bronchial compression/collapse was low for all four radiologists, whereas specificity remained high across the board (Table 3). When both mild–moderate and severe bronchial compression/collapse categories were combined and compared to cases with no compression/collapse, sensitivity showed a slight improvement for Radiologists 1–3 and a marked increase for Radiologist 4. However, this increase in sensitivity was accompanied by a decrease in specificity compared to the results obtained when only severe bronchial compression/collapse was considered against the combination of no and mild–moderate compression/collapse (Table 3).

The number of non-interpretable cases for compression/collapse of the left caudal lobal bronchus was higher than that for the left principal bronchus: six for Radiologist 1, zero for Radiologist 2, two for Radiologist 3, and one for Radiologist 4.

### 3.5. Correlation of Radiographic Variables with Chronic Cough

**Figure 1:**
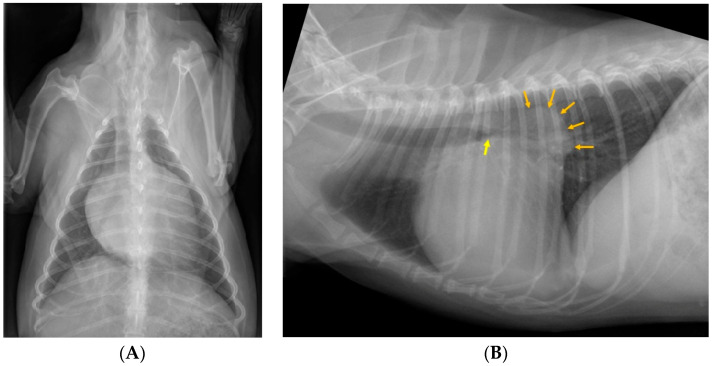
A dorsoventral (**A**) and a left lateral (**B**) thoracic radiograph of a *non-coughing* 7-year-old spayed female Cavalier King Charles Spaniel with severe cardiomegaly and very severe left atrial dilation (orange arrows). All four participating radiologists unanimously thought that the dog was presented with a chronic cough due to left principal (i.e., mainstem) bronchial compression (yellow arrow).

Among the twelve radiographic variables analyzed, no significant correlations with chronic cough were identified by Radiologists 1 and 2. However, Radiologist 3 reported that compression/collapse of the left principal bronchus correlated with chronic cough (odds ratio [OR] 6.06, 95% CI 1.32–39.41, *p* = 0.01). Radiologist 4 reported a similar association for the left caudal bronchus (OR 4.83, 95% CI 1.16–25.00, *p* = 0.02). These isolated findings underscored significant interobserver variability in interpreting bronchial compression/collapse, with no consistent radiographic predictor of cough identified across all evaluators (Figure 1). Multivariable logistic regression models failed to yield additional insights beyond univariate analyses, further highlighting the lack of robust associations.

### 3.6. Correlation Between Presumed Cough and Radiographic Variables

Three of the four radiologists attributed chronic cough primarily to compression/collapse of the left principal bronchus and diffuse bronchial lung pattern (Table 4). Radiologist 4 diverged, emphasizing interstitial pattern in coughing dogs. This discrepancy reinforced the variability in diagnostic prioritization among radiologists, even when evaluating identical radiographic features.

**Figure 2:**
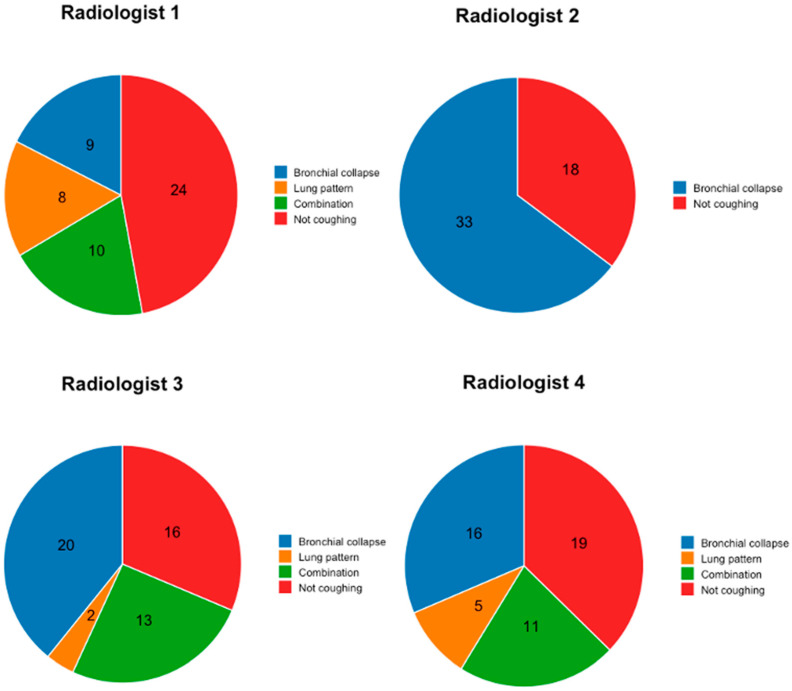
Assumed causes of presumed cough according to the four participating radiologists are shown in pie diagrams. According to Radiologist 1, 27 of the 51 sets of radiographs showed changes that could cause a chronic cough. According to Radiologist 2, there were 33 cases; according to Radiologist 3, this was 35; and according to Radiologist 4, this was 32.

The assumed cause of cough, as reported by the four participating radiologists, is shown in Figure 2.

### 3.7. Interobserver Variability

The interobserver variability among the four participating radiologists showed poor to moderate agreement on the categorical variables (Table 5). On the VHS, the agreement was good, but for the VLAS, it was moderate (Table 6).

According to the preexisting written radiology reports, all made by EBVS-certified radiologists of the Utrecht University, none of the 51 sets of radiographs had an abnormal lung pattern. Table 7 shows the radiographic lung patterns reported by the four participating radiologists. None of the radiologists reported the presence of an alveolar lung pattern.

### 3.8. Intraobserver Variability

We aimed to assess intraobserver variability by offering two sets of radiographs in duplicates, of one non-coughing dog (Dog 1a and 1b) and one dog with chronic cough (Dog 2a and 2b).

Radiologist 2 recognized and reported that both sets were offered twice. For the other three radiologists, minor inconsistencies were detected in their interpretations, which did not affect the conclusions regarding their prediction of coughing status. Only the variables that differ between the observations are shown (Table 8).

## 4. Discussion

This study investigated the relationship between chronic cough and radiographic signs of bronchial narrowing (i.e., compression or collapse) in dogs with cardiomegaly and severe LA dilation secondary to MR. The main conclusion is that subjective radiographic signs of severe left principal bronchial narrowing do not reliably predict chronic cough in dogs with cardiomegaly and LA dilation secondary to severe MR. Our findings show that dogs with and without cough exhibit similar frequencies of severe bronchial narrowing, making radiographic identification of these changes meaningless for predicting cough status.

The reason why we used plain radiographs in this study was that these were readily available for a large number of dogs, and an enlarged heart and a dilated LA can only lead to static airway compression, if any at all. However, because plain radiographs can only show a narrowed bronchial lumen, the exact cause (i.e., compressed by extraluminal pressure from adjacent structures versus collapsed by inherent mural weakness) cannot be revealed; therefore, distinguishing between these two etiologies was impossible.

Contrary to longstanding clinical teaching [1,2,3,4,12,13,14,15,16], our results indicate that radiographic evidence of left principal bronchus narrowing does not reliably distinguish dogs with chronic cough from those without. Both coughing and non-coughing dogs exhibited similar frequencies of severe bronchial narrowing, and radiologists could not accurately predict coughing status based on radiographic findings. Sensitivity and specificity for severe left principal bronchus narrowing as predictors of cough were low, and broadening the criteria to include mild–moderate narrowing improved sensitivity at the expense of specificity, resulting in more false positives. This indicates that broadening the diagnostic criteria improved the ability to detect affected cases but also led to a higher rate of false positives among dogs without cough. Uni- and multivariable regression analyses failed to identify any radiographic variable consistently associated with chronic cough across all radiologists. In line with our findings, previous studies have also challenged the hypothesis that an enlarged LA can compress the left principal bronchus at all [13,24]. In an imaging study on 93 dogs, of which only 2 were coughing, computed tomography revealed focal narrowing of the left principal bronchus in 25 dogs, without any signs of bronchial compression by the LA [24]. Another study on 16 dogs with chronic cough failed to document an association between radiographic and echocardiographic moderate to severe LA enlargement and bronchoscopically detected focal narrowing of the left principal bronchus [13].

Interobserver agreement among the four radiologists in our study was poor to moderate for categorical variables such as bronchial narrowing and lung patterns, consistent with previous reports of suboptimal consistency in thoracic radiographic interpretation [25,26,27,28]. High interobserver variability was especially notable for the assessment of bronchial narrowing, with Radiologist 4 frequently disagreeing with the other three. Despite this variability, all radiologists demonstrated reasonably good interobserver reliability for quantitative measurements (VHS and VLAS), supporting the use of these standardized indices in clinical practice. It is hard to explain what the cause of the large interobserver variation is, when all four radiologists followed the training at the same institution, though at different times. Next to years of experience, mentorship during the training and subjective criteria of the assessed aspects might have played a role.

Administration of medication was not thought to have a confounding effect, as all dogs that were receiving a diuretic, steroid or a cough suppressant were still coughing.

Most radiologists expected a chronic cough in dogs with apparent left main stem (syn. principal) bronchus compression/collapse—a phenomenon described in 1977 for the first time in dogs [4], which subsequently became a belief in clinical teaching [1,2,3,12,13,14,15,16]. However, some dogs with severe radiographic evidence of bronchial narrowing did not cough, while others without such findings did. Since none of the radiologists could reliably predict cough status based on radiographic features, we cannot reject the null hypothesis of our study with confidence. Therefore, we conclude that compression of the left principal or any other lobar bronchi by an enlarged LA is an unlikely cause of chronic cough in dogs with MMVD.

The inability to correlate the radiographic findings with clinical signs challenges the traditional hypothesis that mechanical compression of the left principal bronchus by an enlarged LA is a primary cause of chronic cough in dogs with severe MR. This conclusion is further supported by the observation that some dogs with severe bronchial narrowing did not cough, while others without radiographic evidence of airway narrowing did. These results align with previous studies suggesting that plain radiographs are insufficient for distinguishing between compression due to cardiac enlargement and intrinsic airway disease, such as bronchomalacia [29,30,31,32,33,34]. Though our study did not reveal the mechanism of focal narrowing of the left principal bronchus in dogs with severe LA dilation, we can conclude that chronic cough is not caused by LA enlargement in dogs with severe MR.

Limitations of this study include the retrospective case collection and the inclusion of multiple radiograph sets from the same dogs, which latter may have introduced bias by overrepresenting certain individual characteristics. Additionally, the lack of advanced imaging (such as computed tomography, fluoroscopy, or bronchoscopy) precludes definitive assessment of dynamic airway collapse or concurrent airway pathology. Another limitation of our study is that the radiologists used their own subjective qualitative criteria for assessing the radiographs. Future studies utilizing cross-sectional imaging, artificial intelligence [35,36], objective quantitative criteria, and dynamic assessment techniques may provide further insight into the complex relationship between cardiac enlargement, airway disease, and chronic cough in dogs. 

## 5. Conclusions

In summary, our results suggest that bronchial compression by an enlarged heart and LA is an unlikely cause of chronic cough in dogs with severe MR. The high variability in radiographic interpretation and the lack of correlation between radiographic findings and clinical signs highlight the need for a more nuanced approach to the diagnosis and management of chronic cough in these patients. Practically, our findings reinforce the theory that chronic cough in small breed dogs, even if they have a heart murmur caused by MR, is most likely caused by a primary respiratory disease. This might make the term “cardiac cough” redundant.

## Figures and Tables

**Table 1:** Interpretation of four radiologists (separately for each and combined) for the presence or absence of narrowing (compression/collapse) of the left principal and left caudal lobar bronchus. R = radiologists.

		Left Principal Bronchus		Left Caudal Lobar Bronchus	
	Bronchial Narrowing	Non-Coughing (n = 26)	Coughing (n = 25)	Non-Coughing (n = 26)	Coughing (n = 25)
**Radiologist 1**	no	7	3	23	19
	mild–moderate (<75%)	8	10	1	0
	severe (>75%)	11	12	2	0
	uninterpretable	0	0	0	6
	no + mild–moderate	15	13	24	19
	mild–moderate + severe	19	22	3	0
**Radiologist 2**	no	9	4	20	23
	mild–moderate (<75%)	8	6	4	2
	severe (>75%)	9	15	2	0
	uninterpretable	0	0	0	0
	no + mild–moderate	17	10	24	25
	mild–moderate + severe	17	21	6	2
**Radiologist 3**	no	12	3	19	14
	mild–moderate (<75%)	4	5	3	5
	severe (>75%)	10	17	3	5
	uninterpretable	0	0	1	1
	no + mild–moderate	16	8	22	19
	mild–moderate + severe	14	22	6	10
**Radiologist 4**	no	22	17	13	4
	mild–moderate (<75%)	4	7	9	17
	severe (>75%)	0	0	4	3
	uninterpretable	0	1	0	1
	no + mild–moderate	26	24	22	21
	mild–moderate + severe	4	7	13	20
**Combined R 1–4**	no	52	27	75	60
	no + mild–moderate	74	55	92	84
	mild–moderate + severe	54	72	28	32
	severe	30	44	11	8
**Combined R 1–3**	no	30	10	62	56
	no + mild–moderate	48	31	70	63
	mild–moderate + severe	50	65	15	12
	severe	30	44	7	5

**Table 2:** Sensitivity and specificity of left principal (i.e., mainstem) bronchus narrowing (compression/collapse) as a predictor of cough and its absence as a predictor of no cough for each radiologist separately and combined. CI = confidence interval; R = radiologists.

Left Principal Bronchus	No and Mild–Moderate Versus Severe Narrowing		No Versus Mild–Moderate and Severe Narrowing	
	Sensitivity (95% CI)	Specificity (95% CI)	Sensitivity (95% CI)	Specificity (95% CI)
**Radiologist 1**	48.0 (27.8–68.7)%	57.7 (36.9–76.7)%	88.0 (68.8–97.5)%	26.9 (11.6–47.8)%
**Radiologist 2**	60.0 (38.7–78.9)%	65.4 (44.3–82.8)%	84.0 (63.9–95.5)%	34.6 (17.2–55.7)%
**Radiologist 3**	68.0 (46.5–85.0)%	65.4 (44.3–82.8)%	88.0 (68.8–97.5)%	46.2 (26.6–66.6)%
**Radiologist 4**	0.0 (0.0–14.3)%	100 (86.8–100)%	29.2 (12.6–51.1)%	84.6 (65.1–95.6)%
**Combined R 1–4**	59.5 (47.4–70.7)%	57.4 (48.4–66.0)%	57.1 (48.0–65.9)%	65.8 (54.3–76.1)%
**Combined R 1–3**	59.5 (47.4–70.7)%	60.8 (49.1–71.6)%	56.5 (47.0–65.7)%	75.0 (58.8–87.3)%

**Table 3:** Sensitivity and specificity of left caudal lobar bronchus narrowing (compression/collapse) as a predictor of cough and its absence as a predictor of no cough for each radiologist separately and combined. CI = confidence interval; R = radiologists.

Left Caudal Lobar Bronchus	No and Mild–Moderate Versus Severe Narrowing		No Versus Mild–Moderate and Severe Narrowing	
	Sensitivity (95% CI)	Specificity (95% CI)	Sensitivity (95% CI)	Specificity (95% CI)
**Radiologist 1**	0.0 (0.0–17.7)%	92.3 (74.9–99.1)%	0.0 (0.0–17.7)%	88.5 (69.9–97.6)%
**Radiologist 2**	0.0 (0.0–13.7)%	92.3 (74.9–99.1)%	8.0 (1.0–26.0)%	76.9 (56.4–91.0)%
**Radiologist 3**	20.8 (7.1–42.2)%	92.3 (74.9–99.1)%	41.7 (22.1–63.4)%	76.0 (54.9–90.7)%
**Radiologist 4**	12.5 (2.7–32.4)%	88.5 (69.9–97.6)%	83.3 (62.6–95.3)%	50.0 (29.9–70.1)%
**Combined R 1–4**	42.1 (20.3–66.5)%	52.3 (44.6–59.8)%	53.3 (40.0–66.3)%	55.6 (46.8–64.1)%
**Combined R 1–3**	41.7 (15.2–72.3)%	52.6 (43.8–61.4)%	44.4 (25.5–64.7)%	52.5 (43.2–61.8)%

**Table 4:** Correlation of radiographic variables and the radiologists’ prediction on chronic cough. Only statistically significant variables are shown.

	Accuracy	Variable	Odds Ratio	*p*-Value	95% Confidence Interval
**Radiologist 1**	72% (18/25) correctly predicted as coughing 46% (12/26) correctly predicted as non-coughing	Left principal bronchus narrowing	10.33	<0.01	1.71–114.58
		Bronchial lung pattern	5.16	0.02	1.15–33.06
**Radiologist 2**	72% (18/25) correctly predicted as coughing 42% (11/26) correctly predicted as non-coughing	Left principal bronchus narrowing	56.45	<0.01	6.47–2761.87
		VHS	3.99	0.01	1.54–13.57
		VLAS	13.65	0.01	2.18–132.19
**Radiologist 3**	84% (21/25) correctly predicted as coughing 46% (12/26) correctly predicted as non-coughing	Left principal bronchus narrowing	28.50	<0.01	5.10–232.24
		Right caudal bronchus narrowing	12.13	<0.01	1.54–562.64
		Bronchial lung pattern	Infinite	<0.01	1.83–infinite
		VHS	3.34	0.04	1.24–12.01
		VLAS	21.75	0.01	2.56–341.50
**Radiologist 4**	56% (14/25) correctly predicted as coughing 50% (13/26) correctly predicted as non-coughing	Left caudal bronchus narrowing	4.08	0.04	1.03–18.63
		Interstitial lung pattern	6.52	<0.01	1.55–34.08
		Bronchial lung pattern	11.25	<0.01	2.47–73.88

**Table 5:** Interobserver agreement among the four participating radiologists on categorical radiographic variables. Only statistically significant results are shown. R = radiologist.

Radiologists	Variable	Kappa	*p*-Value
**R1–R2**	Left principal bronchus compression/collapse	0.24	0.02
**R1–R3**	Left principal bronchus compression/collapse	0.21	0.03
	Left caudal lobar bronchus compression/collapse	0.22	<0.01
	Interstitial lung pattern	0.34	<0.01
	Bronchial lung pattern	0.28	0.04
	Prediction of cough	0.35	0.01
	Cause of cough	0.36	<0.01
**R1–R4**	Right caudal lobar bronchus compression/collapse	0.18	0.04
	Cause of cough	0.34	<0.01
**R2–R3**	Left principal bronchus compression/collapse	0.59	<0.01
	Right caudal lobar bronchus compression/collapse	0.20	0.04
	Prediction of cough	0.56	<0.01
**R2–R4**	Right caudal lobar bronchus compression/collapse	0.42	<0.01
	Prediction of cough	0.28	0.04
**R3–R4**	Right cranial lobar bronchus compression/collapse	0.282	0.015
	Interstitial lung pattern	0.20	0.036
	Prediction of cough	0.44	<0.01
	Cause of cough	0.33	<0.01

**Table 6:** Interobserver agreement among the four participating radiologists on VHS and VLAS, using two-way single-score intraclass correlation (ICC).

	ICC	*p*-Value	95% Confidence Interval
VHS	0.76	<0.01	0.65–0.84
VLAS	0.56	<0.01	0.39–0.70

**Table 7:** Number of abnormal lung patterns reported by the four participating radiologists.

	Interstitial	Bronchial
Radiologist 1	3	19
Radiologist 2	1	0
Radiologist 3	12	13
Radiologist 4	34	20

**Table 8:** Intraobserver variability among the radiologists. Dog 1a and 1b represent the same set of radiographs of a non-coughing dog offered twice, while Dog 2a and 2b represent the same set of radiographs of a chronic coughing dog offered twice.

	Dog 1a	Dog 1b	Dog 2a	Dog 2b
**Radiologist 1**	Left principal bronchus was not narrowed	Left principal bronchus collapse (<75%)	Left cranial (caudal) lobar bronchus was not narrowed VHS = 12.5 VLAS = 3.1	Left cranial (caudal) lobar bronchus was uninterpretable VHS = 12.7 VLAS = 2.9
**Radiologist 2**	Reported as duplicate		Reported as duplicate	
**Radiologist 3**	VLAS = 3.0	VLAS = 3.2	Left caudal bronchus collapse (>75%) VLAS = 2.9	Left caudal bronchus collapse (<75%) VLAS = 3.0
**Radiologist 4**	Right medial bronchus was uninterpretable Left cranial (cranial) lobar bronchus was uninterpretable Left cranial (caudal) lobar bronchus was uninterpretable Interstitial lung pattern was not present VHS = 12.2 VLAS = 2.8	Right medial bronchus was not narrowed Left cranial (cranial) lobar bronchus not narrowed Left cranial (caudal) lobar bronchus not narrowed Interstitial lung pattern was present VHS = 12.0 VLAS = 2.5	Left principial bronchus collapse (<75%) Interstitial lung pattern was not present VLAS = 2.9	Left principial bronchus was not narrowed Interstitial lung pattern present VLAS = 3.0

## Data Availability

Data may be provided upon reasonable request.
